# Modelling spatial patterns of correlations between concentrations of heavy metals in mosses and atmospheric deposition in 2010 across Europe

**DOI:** 10.1186/s12302-018-0183-8

**Published:** 2018-12-21

**Authors:** Stefan Nickel, Winfried Schröder, Roman Schmalfuss, Maike Saathoff, Harry Harmens, Gina Mills, Marina V. Frontasyeva, Lambe Barandovski, Oleg Blum, Alejo Carballeira, Ludwig de Temmerman, Anatoly M. Dunaev, Antoaneta Ene, Hilde Fagerli, Barbara Godzik, Ilia Ilyin, Sander Jonkers, Zvonka Jeran, Pranvera Lazo, Sebastien Leblond, Siiri Liiv, Blanka Mankovska, Encarnación Núñez-Olivera, Juha Piispanen, Jarmo Poikolainen, Ion V. Popescu, Flora Qarri, Jesus Miguel Santamaria, Martijn Schaap, Mitja Skudnik, Zdravko Špirić, Trajce Stafilov, Eiliv Steinnes, Claudia Stihi, Ivan Suchara, Hilde Thelle Uggerud, Harald G. Zechmeister

**Affiliations:** 1 Chair of Landscape Ecology, University of Vechta, Vechta, Germany; 2 ICP Vegetation Programme Coordination Centre, Centre for Ecology and Hydrology, Bangor, Gwynedd LL57 2UW UK; 3 Moss Survey Coordination Centre, Frank Laboratory of Neutron Physics, Dubna, Moscow Region Russian Federation; 4 Ss. Cyril and Methodius University, Skopje, Macedonia; 5 National Botanical Garden, Academy of Science of Ukraine, Kiev, Ukraine; 6 University of Santiago de Compostela, Santiago de Compostela, Spain; 7 Sciensano, Tervuren, Belgium; 8 Ivanovo State University of Chemistry and Technology, Ivanovo, Russia; 9 Dunarea de Jos University of Galati, Galati, Romania; 10 Norwegian Meteorological Institute, Oslo, Norway; 11 W. Szafer Institute of Botany, Polish Academy of Sciences, Kraków, Poland; 12 Meteorological Synthesizing Centre East, Moscow, Russia; 13 TNO, Utrecht, The Netherlands; 14 Jožef Stefan Institute, Ljubljana, Slovenia; 15 University of Tirana, Tirana, Albania; 16 National Museum of Natural History, Paris, France; 17 Tallinn Botanic Garden, Tallinn, Estonia; 18 Institute of Landscape Ecology, Slovak Academy of Sciences, Bratislava, Slovak Republic; 19 University of La Rioja, Logroño, Spain; 20 Natural Resources Institute Finland (Luke), Oulu, Finland; 21 Valahia University of Targoviste, Targoviste, Romania; 22 University of Vlora, Vlorë, Albania; 23 University of Navarra, Navarra, Spain; 24 Slovenian Forestry Institute, Ljubljana, Slovenia; 25 Green Infrastructure Ltd., Zagreb, Croatia; 26 Norwegian University of Science and Technology, Trondheim, Norway; 27 Silva Tarouca Research Institute for Landscape and Ornamental Gardening, Průhonice, Czech Republic; 28 Norwegian Institute for Air Research, Kjeller, Norway; 29 University of Vienna, Vienna, Austria

**Keywords:** Biomonitoring, Chemical transport models, Correlation analysis, Ecological classification, Linear discriminant analysis, Logistic regression

## Abstract

**Background:**

This paper aims to investigate the correlations between the concentrations of nine heavy metals in moss and atmospheric deposition within ecological land classes covering Europe. Additionally, it is examined to what extent the statistical relations are affected by the land use around the moss sampling sites. Based on moss data collected in 2010/2011 throughout Europe and data on total atmospheric deposition modelled by two chemical transport models (EMEP MSC-E, LOTOS-EUROS), correlation coefficients between concentrations of heavy metals in moss and in modelled atmospheric deposition were specified for spatial subsamples defined by ecological land classes of Europe (ELCE) as a spatial reference system. Linear discriminant analysis (LDA) and logistic regression (LR) were then used to separate moss sampling sites regarding their contribution to the strength of correlation considering the areal percentage of urban, agricultural and forestry land use around the sampling location. After verification LDA models by LR, LDA models were used to transform spatial information on the land use to maps of potential correlation levels, applicable for future network planning in the European Moss Survey.

**Results:**

Correlations between concentrations of heavy metals in moss and in modelled atmospheric deposition were found to be specific for elements and ELCE units. Land use around the sampling sites mainly influences the correlation level. Small radiuses around the sampling sites examined (5 km) are more relevant for Cd, Cu, Ni, and Zn, while the areal percentage of urban and agricultural land use within large radiuses (75–100 km) is more relevant for As, Cr, Hg, Pb, and V. Most valid LDA models pattern with error rates of < 40% were found for As, Cr, Cu, Hg, Pb, and V. Land use-dependent predictions of spatial patterns split up Europe into investigation areas revealing potentially high (= above-average) or low (= below-average) correlation coefficients.

**Conclusions:**

LDA is an eligible method identifying and ranking boundary conditions of correlations between atmospheric deposition and respective concentrations of heavy metals in moss and related mapping considering the influence of the land use around moss sampling sites.

**Electronic supplementary material:**

The online version of this article (10.1186/s12302-018-0183-8) contains supplementary material, which is available to authorized users.

## Background

The United Nations Economic Commission for Europe (UNECE) Convention on Long-range Transboundary Air Pollution (CLRTAP) of 1979 and its eight protocols are aimed at limiting and reducing air pollutants. Under the LRTAP convention, the European monitoring and evaluation programme (EMEP) gathers information on emission from its parties, collects data on air and precipitation quality and models atmospheric transport and deposition of air pollutants [1]. Beyond this, biomonitoring programmes provide data on concentrations in various biological matrices potentially correlated with atmospheric deposition of heavy metals (HM). Within the LRTAP convention, European Moss Survey (EMS) is conducted using naturally growing mosses as biomonitors of atmospheric deposition of air pollutants. Since 1990, moss specimens have been sampled every 5 years at up to 7300 sampling sites in up to 35 countries [2–4] to determine the concentrations of heavy metals (HM), nitrogen (N, since 2005) and persistent organic pollutants (POPs, since 2010) [4, 5]. The EMS is coordinated by the ICP Vegetation, an international cooperative programme (ICP) reporting on impacts of air pollution on vegetation to the LRTAP convention [3].

Based on EMS data from 2005, atmospheric deposition has been identified as the main factor determining the spatial variation of concentrations of cadmium (Cd) and lead (Pb) in moss specimens collected throughout Europe [6–8]. Harmens et al. [9] found significant correlations between Cd and Pb concentration in moss and respective atmospheric deposition modelled by EMEP for more than two-thirds of the countries participating in the European Moss Survey. Schröder et al. [10] correlated Cd, mercury (Hg), and Pb concentrations in deposition and moss data from the EMS 2005 within a spatial framework of ecologically defined land classes by use of the numeric chemical transport model (CTM) of EMEP MSC-East [11]. In further studies, also land use around the sampling sites is shown to be an important factor affecting element concentrations in moss [12–14].

The above-mentioned findings were verified in the investigation presented in this paper using data collected in the EMS 2010. The present study addresses the following objectives.

1. *Correlation analysis* Examination of correlations between concentrations of HM in moss from the EMS 2010/2011 and respective atmospheric deposition as modelled by use of the CTMs EMEP MSC-East and LOTOS EUROS (LE), and to which extent the correlations are specific for ecological land classes of Europe (ELCE) [15].
2. *Statistical modelling* Calculation, to which extent the amount of ELCE-specific correlation coefficients is affected by the areal percentage of land use around the sampling sites potentially indicating influences of local emission sources as for instance agricultural and urban land use or point sources of air pollutants. Hence, the reason for different ELCE-specific correlation coefficients was investigated.
3. *Predictive mapping* Land use-dependent predictions and mapping of correlation patterns across Europe (site-related/area-related) and, finally, aggregation of predicted spatial patterns for decision support (e.g. moss survey network planning).

For this investigation, data on atmospheric deposition of HM derived from the EMEP MSC-East [11] were supplemented by deposition data calculated by use of the chemical transport model LOTOS-EUROS (LE) [16].

## Methods

Data on element concentration in moss were correlated with respective modelled atmospheric deposition specifically for ecological land classes of Europe (ELCE) and major land use categories around the sampling sites derived from CORINE land cover 2006 and Global land cover 2000 [17, 18] (Table 1).

**Table 1:** Data used for statistical analysis

Data	Comment and source	Unit
Element concentration in moss	As, Cd, Cr, Cu, Hg, Ni, Pb, V, and Zn conc. in moss from the European Moss Survey 2010/2011	μg/g
Atmospheric deposition	Modelled total deposition of As, Cd, Cr, Cu, Ni, Pb, V, Zn summed over 3 years (LOTOS-EUROS 2009–2011, [21])	µg/m^2^
	Modelled total atmospheric deposition of Cd, Hg, Pb (EMEP MSC-East) summed over 3 years (EMEP 2008–2010)^a^	µg/m^2^
ELCE_40_	Ecological land classes of Europe [15]	40 land classes
Spatial density of land use around moss sampling sites	Areal percentage of urban, agricultural, and forestry land use, each within a 1, 5, 10, 25, 50, 75, and 100 km radius around the moss sampling sites, derived from CORINE land cover 2006 [17] and global land cover 2000 [18] for Russia, Ukraine and Belarus	%

^a^HM data provided by MSC-East (November 2013)

### Data on element concentrations in moss

In 2010/2011, moss specimens were collected at 4499 sample sites in 26 countries across Europe following a standardized experimental protocol [19]. Further countries like Germany, Ireland and United Kingdom who participated in former moss surveys did not participate in 2010. To provide field-based evidence of the extent of long-range transboundary pollution in Europe the monitoring sites are located in background areas, e.g. sampling sites were at least 300 m away from major roads and 100 m away from any road or houses. Primarily, *Pleurozium schreberi* (Brid.) Mitt., *Hylocomium splendens* (Hedw.) Schimp., *Hypnum cupressiforme* Hedw. s.str. and *Pseudoscleropodium purum* (Hedw.) M. Fleisch (synonym *Scleropodium purum* Hedw. Limpr.) [20] were sampled, but also 32 other species (7% of the samples). For each site, at least five individual moss samples of the same species were collected. Only the 2- to 3-year-old shoots of the mosses were used for the analyses. Concentrations of nine HMs: arsenic (As), cadmium (Cd), chromium (Cr), copper (Cu), mercury (Hg), nickel (Ni), lead (Pb), vanadium (V), and zinc (Zn) were determined [4, 9].

### Data on atmospheric deposition

Statistical relations between element concentrations in moss and atmospheric deposition of HM derived from the numeric chemical transport models (CTMs) LOTOS-EUROS (LE) [16, 21] and EMEP [11] were examined. CTMs are based on mathematical descriptions of relevant physical and chemical processes in the atmosphere and are mostly used for large-scale, area-wide estimates of atmospheric deposition [21]. The accuracy of deposition modelling basically depends on the quality of the input data (emission, meteorology, land use, other conditions) used for modelling atmospheric transport and deposition processes as well as intrinsic model uncertainties.

The EMEP deposition data were supplied by the meteorological synthesizing centres MSC-East (Moscow) of EMEP operating under the LRTAP convention. Travnikov and Ilyin [11] used emission data to calculate atmospheric deposition of Cd, Hg, and Pb. To verify these model calculations, the results were compared to Cd and Pb measurement data from up to 66 EMEP sites and to Hg data collected at up to 22 EMEP sites [22]. The verified model results were then mapped on grids of 50 km × 50 km [11].

Following Harmens et al. [9], in this investigation the 3-year sum of HM deposition modelled by EMEP (on a 50 km by 50 km grid) corresponds to the HM concentration in the sampled 3-year-old shoots of the mosses. Here, the deposition data from 2008 to 2010 was assigned to the data collected in EMS 2010/2011. The 3-year sums of deposition 2009–2011 from LE [16, 21] were assigned to the concentrations of HM in moss collected in EMS 2010/2011. LE provides deposition rates of As, Cd, Cr, Cu, Ni, Pb, V, and Zn on a 25 km by 25 km grid covering Europe. Additional information about the CTM is given in Additional file 1: Table S2.

### Ecological land classification of Europe

The data on element concentrations in moss and atmospheric deposition were spatially joined to the map of ecological land classes of Europe (ELCE) (Additional file 1: Figure S1, Table S1) derived from Hornsmann et al. [15]. According to the level of spatial differentiation, the ecological classification encompasses 40 (ELCE_40_) to 200 (ELCE_200_) classes identified by 48 geo-data layers on potential natural vegetation [23], altitude above sea level [24], soil texture [25], and monthly averages of precipitation and air temperature (1961–2002) [26]. ELCE_40_ and ELCE_200_ were calculated and mapped by means of classification and regression trees [27]. To ensure the best possible compliance with minimum sample size specified for each ecoregion [28, 29], ELCE_40_ was used, whereby ELCE units occurring sporadically and with a total spatial extent below 4.2% were summarized to one class (“others”).

### Statistical analysis

#### Correlation analysis

**Fig. 1:**
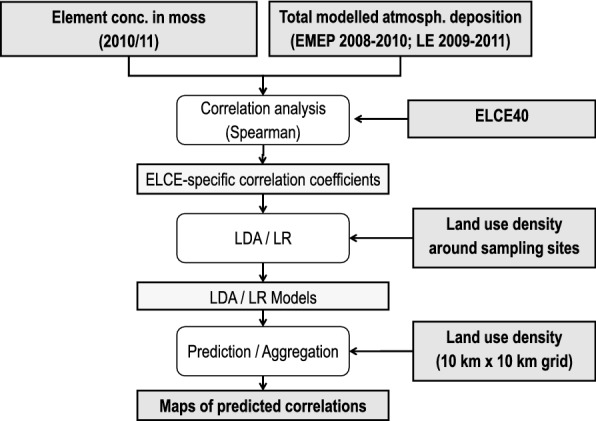
Design of statistical analysis (*LDA* linear discriminant analysis, *LR* logistic regression)

The statistical design comprises the calculation of Spearman rank correlation coefficients (*r*_s_) for quantifying the relation between concentrations in mosses and modelled atmospheric deposition of HM and N (Fig. 1). The measured concentrations of As, Cd, Cr, Cu, Hg, Ni, Pb, V, and Zn in moss were correlated with respective total atmospheric deposition data as modelled by EMEP and LE. Thereby, ecological land classes (ELCE_40_) within participating European countries were used as coding variable for calculating ELCE-specific correlations. Due to a non-normal distribution in most of the subsamples, Spearman rank correlation coefficients (*r*_s_) were determined. The correlation coefficients were classified according to Brosius [30] as very weak (< 0.2), weak (0.2–0.4), moderate (0.4–0.6), strong (0.6–0.8), and very strong (> 0.8).

#### Linear discriminant analysis/logistic regression

The second step is to investigate the reason for the difference of ELCE-specific correlations. LDA models were used to find linear separation lines as best discriminate of sampling sites in ELCE regions revealing high or low element-specific correlation coefficients. LDA attempts to find a multivariate discriminant function *Y* = *b*_0_ + *b*_1_*X*_1_ + *b*_2_*X*_2_ +  ⋯ describing a linear combination of two or more predictors (*X*_1_, *X*_2_, …) and respective coefficients (*b*_0_, *b*_1_, *b*_2_, …). The aim is to separate groups of data in a scatterplot so that the variation in data within each group is minimized [31–33] and express the contribution of each predictor in the selected discriminant model. For binary classification of ELCE and their allocated sampling sites showing high (= A) or low (= B) correlations, medians of the ELCE-specific Spearman coefficients were taken for defining element-specific class boundaries between high and low correlation levels. ELCE with coefficients above the class boundaries in terms of element-specific medians were classified as ‘A’ and ELCE below the class boundaries as ‘B’. Twenty-one variables for spatial density of agricultural, forestry, and urban land use within a 1, 5, 10, 25, 50, 75, and 100 km radius around the sampling sites (Table 1) were taken as potential predictors for HM concentrations in moss samples. As the target variable is already determined by atmospheric deposition, it was not considered as a predictor. Further potential influencing factors like elevation, precipitation, population density as investigated by Nickel et al. [34] were examined in a pre-analysis using LDA, but were excluded due to low relevance. Since the values of each of these predictors range between 0 and 100%, data did not need to be standardized as recommended for LDA by Schönwiese [35]. Overall, twelve LDA models were built with regard to available EMEP deposition values for (Cd, Pb, Hg) and LOTOS-EUROS deposition estimations for (As, Cd, Cr, Cu, Ni, Pb, V, and Zn). It was examined whether the variance could be sufficiently explained by just two of the potential 21 linear discriminants (= spatial density [%] of urban, agricultural, and forestry land use, each within a 1, 5, 10, 25, 50, 75, and 100 km radius around the moss sampling sites, Table 1) to keep the models as simple as possible and allowing for a better interpretation and visualization of the results. Here, near-zero coefficients (linear combination coefficient ranges between − 1 and 1) and correlated predictors have been removed to avoid multicollinearity. For example, if the coefficient of urban land use within a 10 km radius was closer to zero than the 5 km coefficient, the latter was taken.

Logistic regression (LR) is similar to LDA, as it also explains a categorical variable by the values of continuous independent variables. LR is preferable in applications where the independent variables are not normally distributed. Since LR is less concrete, LDA in the present study was used for model building and LR for verification of LDA results.

#### Predictions

LDA models were firstly applied on the Europe-wide dataset of moss sampling sites with information on land use density around the sampling sites. Model-specific error rates (%) were calculated by means of confusion matrix values (actual vs. predicted values). Charts for the linear discriminant functions were used for plausibility checks. Logistic regression models were built using the same predictors from the LDA models. Confusion matrices and error rates (%) specified for each LR model were calculated and compared with the statistical characteristics of the LDA models.

To verify to which extent the models really separate sampling sites showing high or low correlations between element concentrations in moss and respective atmospheric deposition, bivariate Spearman coefficients for the correlations between element concentrations in moss and atmospheric deposition were again calculated for the following subsamples: sampling sites located within all ELCE classes, ELCE classes showing correlations above and below the element-specific class boundaries between high and low correlation levels defined in Table 1. Each subsample was further divided into groups of sampling sites classified by LDA into category A or B. The more B sampling sites modelled by LDA show low or, vice versa, A sites reveal high correlations, the more efficient the between-class separation through the modelling and thus the relevance of predictors.

Geographic information on the spatial density of agricultural, forestry, and urban land use within a 1, 5, 10, 25, 50, 75, and 100 km radius around the sampling sites available with blanket coverage of Europe was taken as predictors for estimating categories of correlations (A, B) between atmospheric deposition of nine HM in Europe using LDA models and to transform spatial information on the land use to spatial correlation patterns across Europe. Finally, spatial patterns estimated by the best LDA models were aggregated by calculating the number of element-specific A classifications (= above element-specific class boundaries between high and low correlation levels as defined in Table 2) to reduce complexity which is more appropriate for decision support. All statistical analyses were performed using R programming language [36], in particular functions for LDA as implemented in the ‘MASS’ package extending R’s core functionality [37].

**Table 2:** Correlations between element concentrations in moss and modelled atmospheric deposition specified for ecological land classes of Europe

ELCE_40_	EMEP			LOTOS-EUROS							
	Cd	Hg	Pb	As	Cd	Cr	Cu	Ni	Pb	V	Zn
All	0.65** (3777)	0.14** (3313)	0.70** (3604)	0.30** (3274)	0.65** (3633)	0.03** (3820)	0.50** (3465)	0.09** (3772)	0.64** (3490)	0.19** (3832)	0.17** (3965)
B_1	0.39** (73)	0.48** (67)	0.54** (73)	*0.23 (67)*	0.45** (73)	− 0.13 (73)	0.22 (73)	− 0.24 (73)	0.67** (73)	− 0.09 (73)	0.29* (73)
B_2	0.51** (110)	− 0.18 (110)	0.63** (110)	− 0.11 (111)	0.17 (110)	− 0.52** (111)	− 0.20 (110)	− 0.67** (110)	0.27 (110)	*0.13 (34)*	0.09 (111)
C_0	0.52** (253)	0.19** (239)	0.62** (252)	0.45** (246)	0.52** (253)	0.16* (258)	0.51** (252)	− 0.04 (258)	0.65** (252)	0.27** (227)	0.11 (259)
D_7	0.13 (186)	0.29** (135)	− 0.01 (186)	− 0.14 (134)	0.19* (186)	0.22** (186)	0.05 (186)	− 0.23** (186)	0.25** (186)	− 0.54** (186)	− 0.04 (186)
D_8	0.37* (42)	− 0.10 (34)	0.49** (42)	*0.10 (34)*	0.31* (42)	− 0.10 (42)	0.52** (42)	− 0.20 (42)	0.44** (42)	0.07 (42)	− 0.03 (42)
D_10	− 0.08 (11)	*0.20 (11)*	0.22 (11)	*0.05 (11)*	− 0.23 (11)	− 0.11 (11)	0.08 (11)	− 0.25 (11)	0.12 (11)	− 0.27 (11)	0.09 (11)
D_13	0.72** (99)	*0.18 (76)*	0.61** (99)	− 0.26** (114)	0.74** (99)	− 0.48** (139)	0.40** (99)	− 0.21* (121)	0.44** (255)	− 0.20* (156)	0.10 (139)
D_14	0.39** (82)	0.43** (77)	0.46** (78)	*0.15 (83)*	0.57** (82)	− 0.2* (119)	0.36** (94)	− 0.04 (119)	0.55** (78)	− 0.21* (136)	0.33** (137)
D_17	− 0.03 (115)	0.12** (71)	0.36** (89)	0.46** (127)	0.29** (115)	0.69** (147)	0.46** (93)	0.33** (148)	0.40** (89)	0.19* (153)	0.17 (154)
D_18	0.22** (255)	0.14* (248)	0.29** (255)	0.47** (201)	0.33** (255)	*0.08 (255)*	0.37** (255)	0.43** (255)	0.44** (255)	0.22** (253)	0.21** (255)
D_19	0.47** (258)	0.18* (165)	0.54** (258)	0.24** (165)	0.47** (258)	*0.06 (258)*	0.39** (258)	*0.03 (258)*	0.53** (258)	0.27** (258)	0.19** (258)
D_22	0.21** (168)	0.09 (166)	0.36** (168)	*0.06 (167)*	0.29** (168)	0.43** (171)	0.29** (168)	*0.04 (171)*	0.42** (168)	0.28** (171)	0.05 (171)
F1_1	0.72** (87)	0.37** (87)	0.62** (87)	0.57** (42)	0.76** (87)	*0.06 (95)*	0.48** (87)	− 0.01 (94)	0.65** (87)	− 0.14 (91)	0.39** (95)
F1_2	0.17** (308)	− 0.15** (308)	0.38** (192)	− 0.19** (289)	0.20** (308)	− 0.11 (191)	0.31** (191)	− 0.07 (192)	0.43** (192)	*0.14 (191)*	*0.17 (154)*
F2_5	0.53** (66)	*0.19 (65)*	0.33** (66)	− 0.51 (12)	0.72** (66)	− 0.12 (77)	0.27* (66)	− 0.11 (71)	0.40** (66)	0.01 (37)	0.38** (77)
F2_6	0.41** (264)	0.01 (238)	0.48** (264)	− 0.18** (250)	0.46** (264)	− 0.39** (301)	0.31** (264)	− 0.49** (291)	0.28** (264)	− 0.14* (307)	0.18** (301)
F3_1	0.26** (201)	0.20** (189)	0.35** (201)	0.24** (173)	0.43** (201)	− 0.09 (204)	0.13 (201)	− 0.09 (203)	0.30** (201)	− 0.05 (201)	0.20** (204)
F3_2	0.53** (115)	− 0.21* (113)	0.45** (115)	*0.17 (115)*	0.53** (115)	*0.13 (114)*	0.13 (114)	*0.17 (115)*	0.47** 5 (115)	0.08 (114)	*0.17 (114)*
F4_1	0.28 (17)	0.57* (17)	0.51* (17)	− 0.41 (11)	0.09 (17)	*0.30 (17)*	− 0.12 (17)	− 0.27 (17)	0.02 (17)	*0.36 (17)*	0.03 (17)
F4_2	0.53** (468)	− 0.06 (394)	0.56** (468)	− 0.09* (491)	0.46** (468)	− 0.45** (540)	0.00 (467)	− 0.14** (510)	0.01 (468)	− 0.01 (571)	− 0.08 (541)
G1_0	0.20* (126)	0.03 (63)	0.13 (126)	0.02 (137)	0.14 (126)	− 0.04 (189)	0.01 (126)	− 0.12 (189)	0.10 (126)	− 0.17* (177)	0.29** (189)
G2_0	0.08 (186)	0.21** (174)	0.26** (162)	− 0.12 (174)	0.32** (186)	− 0.49** (152)	0.36** (151)	0.41** (162)	0.40** (162)	0.03 (152)	− 0.14 (176)
J_2	0.43** (60)	0.50** (60)	0.51** (59)	*0.02 (60)*	0.54** (60)	*0.25 (10)*	0.49** (49)	0.60** (59)	0.55** (59)	*0.21 (49)*	− 0.16 (50)
S_0	0.69** (54)	− 0.04 (44)	0.58** (54)	*0.13 (41)*	0.64** (54)	*0.18 (61)*	0.37* (55)	− 0.23 (61)	0.62** (54)	− 0.15 (55)	0.42** (62)
U_1	0.47** (47)	0.05 (47)	0.51** (47)	0.72** (24)	0.57** (47)	*0.23 (49)*	0.49** (47)	− 0.09 (49**)**	0.48** (47)	0.31* (49)	0.35* (49)
U_2	− 0.01 (81)	− 0.06 (73)	0.13** (80)	− 0.24* (98)	0.16 (81)	− 0.45** (102)	0.32** (79)	− 0.01 (98)	0.18 (80)	− 0.24* (102)	0.02 (103)
Others	0.78** (45)	0.09 (42)	0.74** (45)	0.37* (40)	0.79** (45)	− 0.01 (53)	0.17 (45)	− 0.35* (53)	0.68** (45)	*0.10 (50)*	0.39** (53)
Class boundary	0.35	0.10	0.43	0.00	0.44	0.05	0.32	0.00	0.40	0.10	0.15

EMEP/LOTOS-EUROS = chemical transport models used for calculating atmospheric deposition; ELCE_40_ = ecological land classes of Europe [15] and other ELCE which were summarized to one class (“others”); correlation coefficients according to Spearman (*p < 0.05, **p < 0.01); (*n*) in brackets = sample size; ELCE-specific correlations above the element-specific class boundary between low and high correlation levels (= category A) are in italic print

## Results

### Correlations between HM concentrations in moss and atmospheric deposition for ELCE categories across Europe and for Europe as a whole

All analyses with HM concentration in moss were based on a reasonably large sample size of at least 3274 (As) out of 3965 (Zn) sample points. The minimum sample sizes for elements and ELCE_40_ classes were calculated and presented by Schröder et al. [28, 29]. As the number of moss sampling sites was very low (> 10 in the classes D_16, D_21, L_2, M_5, and M_6), the correlations for these classes are not considered reliable and are not described below. However, these four classes altogether represent only 2.3% (= 69,600 km^2^) of the sampled area in the countries participating in the EMS (= 3,083,500 km^2^).

#### Cadmium

**Fig. 2:**
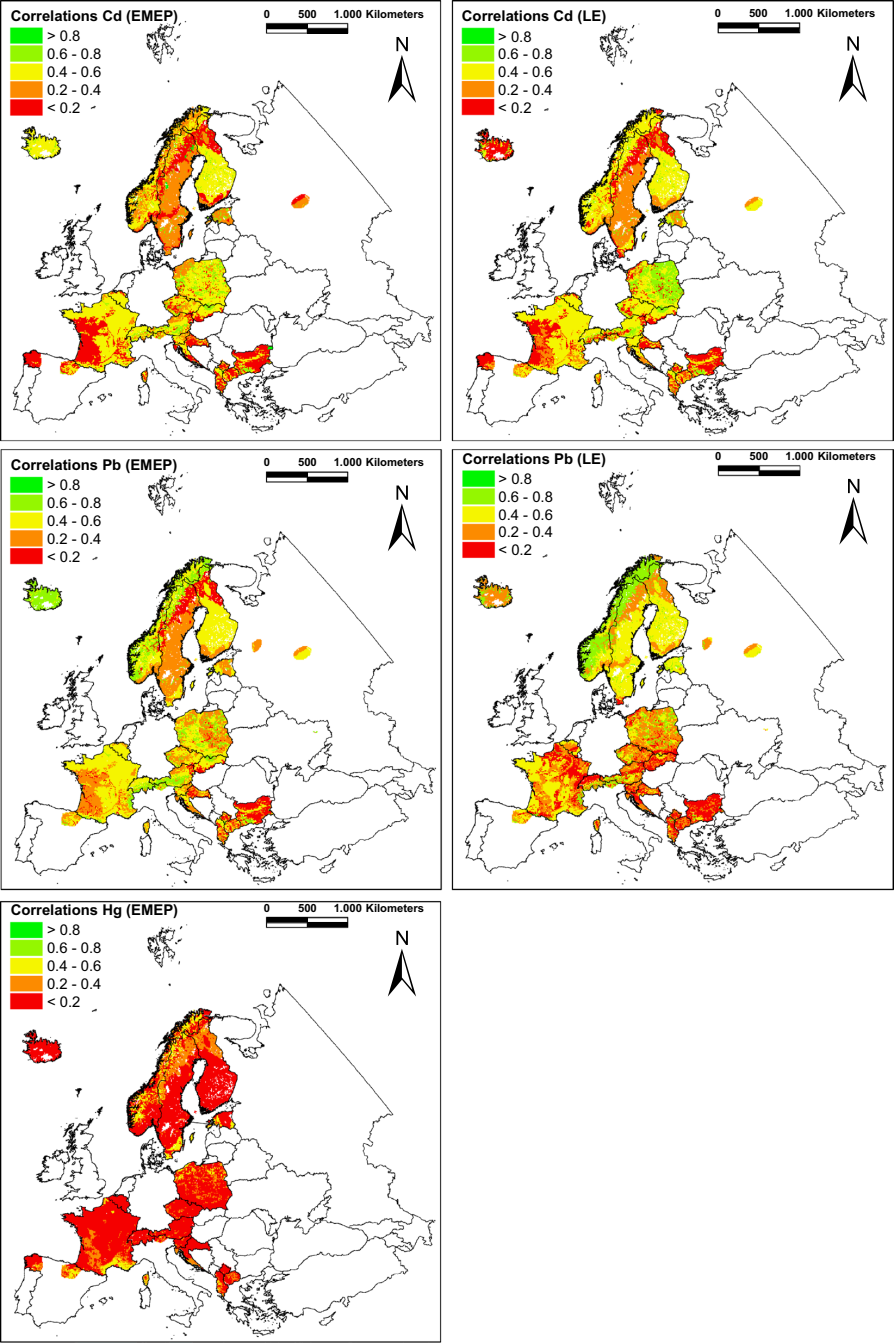
ELCE-specific correlations of Cd, Pb and Hg concentrations in mosses and respective modelled atmospheric deposition. Atmospheric deposition was modelled by LE (2009–2011) or EMEP (2008–2010); concentration values in mosses were determined in 2010

Strong correlations between element concentrations measured in moss and modelled deposition (EMEP, LE) with coefficients (*r*_s_) ranging from 0.6 to 0.8 were achieved for 7% (EMEP) up to 10.5% (LE) of the area of ELCE_40_ coverage of all countries participating in the EMS 2010 together (Table 2). These ELCE_40_ categories (D_13, F1_1, S_0, and “others”) are located in Poland, Switzerland and Austria (Fig. 2). The strength of the Europe-wide correlation is also high (*r*_s_ = 0.65, *p* < 0.01). Moderate r_s_ values occurred for ELCE_40_ units covering 47.0–49.6% of the landmass. Moss data from each 5 ELCE_40_ (for LE in parts other than for EMEP) are weakly correlated with the modelled Cd deposition (EMEP: 15.0%; LE: 17.5%). 7 (EMEP) up to 9 (LE) out of 27 ELCE_40_ units reveal non-significant or very weak correlations (23.5–31.1%).

#### Lead

For Pb, in 4 (EMEP) up to 6 (LE) out of 27 ELCE_40_ units the *r*_s_ values were not significant (12.0–20.3% of area of ELCE units covered by moss sampling sites) (Table 2). From the remaining ELCE_40_ classes, 6 (in case of LE, 26.3%) and 7 (EMEP, 28.9%) ELCE units show Spearman’s rank coefficients between 0.2 and 0.4. For another 10 ELCE categories (LE) and, respectively, 11 ELCE categories (EMEP), correlation coefficients came out to be between 0.4 and 0.6. The area comprises 36.6–43.5% of the total area covered by moss samples mainly located in Finland, Sweden and France (Fig. 2). Highest correlations (0.6 > *r*_s_ > 0.8) were found for max. 5 ELCE classes: D_13 (only EMEP), S_0 (only LE), B_2, C_0, F1_1, “others” (both EMEP and LE) (15.6–16.8% of the landmass) predominately distributed in Norway. With regard to the samplings across Europe, Spearman’s rank coefficients are 0.64 (LE) and 0.7 (EMEP).

#### Mercury

For Hg, in 21 out of 27 ELCE units, the *r*_s_ values were not significant, below 0.02 or even negative (82.5% of area of ELCE classes covering all participating countries together). It is clear that the correlation between moss data from the EMS 2010 and EMEP modelled deposition is very low (*r*_s_ = 0.14, Table 2). Above-average correlations with coefficients between 0.4 and 0.6 were only found for ELCE units B_1, D_14, F4_1, and J_2 (9.2% of the area), sparsely located in Fennoscandia, Estonia, Poland, France and Spain (Fig. 2). For another 2 ELCE classes (D_7, F1_1), correlation coefficients were between 0.2 and 0.4, comprising 8.3% of the area covered by moss samples.

#### Arsenic

**Fig. 3:**
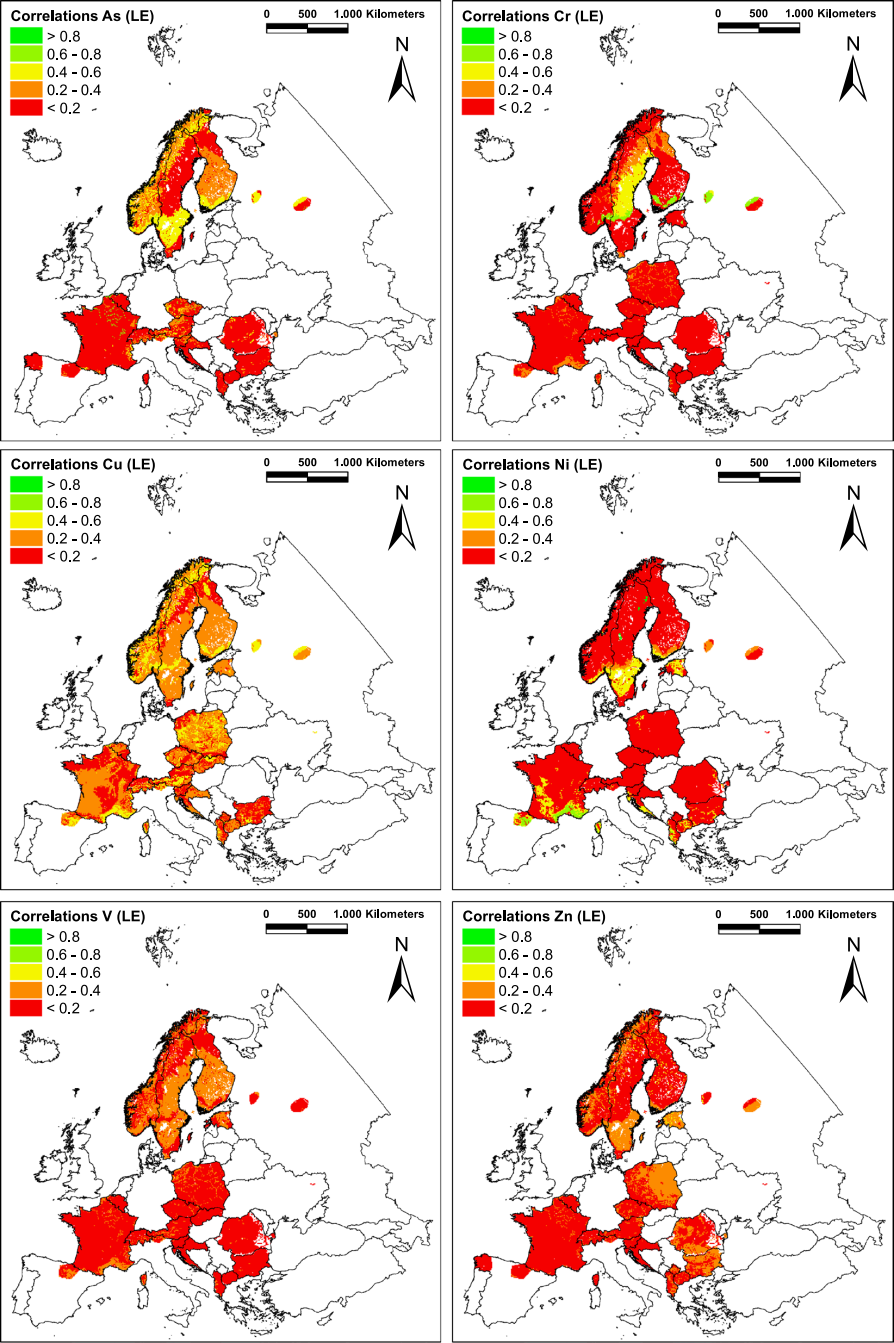
ELCE-specific correlations of As, Cr, Cu, Ni, V and Zn concentrations in mosses and respective modelled atmospheric deposition. Atmospheric deposition was modelled by LE (2009–2011) or EMEP (2008–2010); concentration values in mosses were determined in 2010

For Europe as a whole, low correlations between As concentrations in moss and respective modelled atmospheric deposition (LOTOS-EUROS) were found (*r*_s_ = 0.3). 14 out of 27 ELCE_40_ units reveal non-significant correlations within 41.1% of the sampled ELCE_40_ area (Table 2). In 5 out of the remaining 13 ELCE_40_ units, variables were negatively correlated (30.1%). Three ELCE_40_ classes reveal significant weak correlations with *r*_s_ values between 0.2 and 0.4 (13.1%). Merely 4 ELCE_40_ units show moderate coefficients between 0.4 and 0.6 (C_0, D_17, D18, and F1_1). The ELCE_40_ unit with the highest correlation was U_1 (*r*_s_ = 0.72, *p* < 0.01) comprising dispersed small areas within the participating countries (1%) (Fig. 3).

#### Chromium

Of all elements examined, Cr reveals the weakest Europe-wide correlation between concentrations in moss and total deposition modelled by LE (*r*_s_ = 0.03, Table 2). For 16 out of 27 ELCE_40_ units, the r_s_ values were not significant (49.5% of area of ELCE units covered by moss sampling sites), and for 37.3%, the r_s_ values were below 0.02 or even negative (Fig. 3). A strong correlation (*r*_s_ = 0.69) could be shown for land class D_17, covering 2.4% of the analysed area, located in Sweden, Finland and Russia. D_22 (5.4%) as a part of Sweden reveals at least moderate correlations (*r*_s_ = 0.43). The remaining surface showing low correlations is allocated to ELCE unit D_7, which covers 5.4% of the landmass.

#### Copper

The largest area covered by moss sampling sites (48.9%) is allocated to low correlations (*r*_s_) between 0.2 and 0.4. Moderately strong correlations were found for 6 out of 27 ELCE_40_ units (C_0, D_8, D_17, F1_1, J_2, and U_2) sparsely distributed in almost every participating country and comprising 15.6% of the ELCE units. In comparison, Europe as a whole is also characterized by an intermediately strong correlation (*r*_s_ = 0.5). All other 10 out of 27 ELCE_40_ units reveal non-significant correlations within 35.5% of the sampled ELCE_40_ area.

#### Nickel

For Ni, most of the ELCE_40_ units reveal negative correlations (28.5% of the analysed area) or non-significant values (53.8%). Significant positive correlations in ELCE_40_ classes were found for D_17 (0.2 > *r*_s_ > 0.4), D_18, G2_0 (0.4 > *r*_s_ > 0.6) and J_2 (0.6 > *r*_s_ > 0.8) (Table 2). Together, these four land classes comprise only 13.1% of the ELCE territory within participating countries, in particular Sweden, Estonia and France (Fig. 3). Overall, this corresponds to a very low correlation of *r*_s_ = 0.09 across Europe.

#### Vanadium

With respect to atmospheric V deposition modelled by LE and respective concentration in moss, merely 5 of 31 ELCE_40_ classes (plus “others”) reveal significant positive, low Spearman’s rank coefficients (C_0, D_18, D_19, D_22, and U_1). They cover 25.7% of the sampled area and can be primarily found in Fennoscandia, northern Spain and France (Fig. 3). 2.4% of the area analysed (D_17) shows significant low correlations. The remaining ELCE units (66.8%) reveal non-significant or negative correlations (Table 2). For V across Europe, the Spearman coefficient also has to be classified as low and amounts to *r*_s_ = 0.19.

#### Zinc

On the European level, the correlation between modelled Zn deposition (LE) and concentrations in moss is significantly low with *r*_s_ = 0.17. The only ELCE_40_ unit with an intermediately high correlation is S_0, located in parts of Estonia, Finland and Russia (1.4% of the sampled area). The 7 out of 26 ELCE_40_ classes with at least low correlations were the following: D_18, F1_1, F2_5, F3_1, G1_0, U_1, and “others”, located in eastern and northern parts Europe. The coefficients for the remaining ELCE units are very low or non-significant (29.5% and 45.6% of the landmass).

### Linear discriminant analysis/logistic regression

**Fig. 4:**
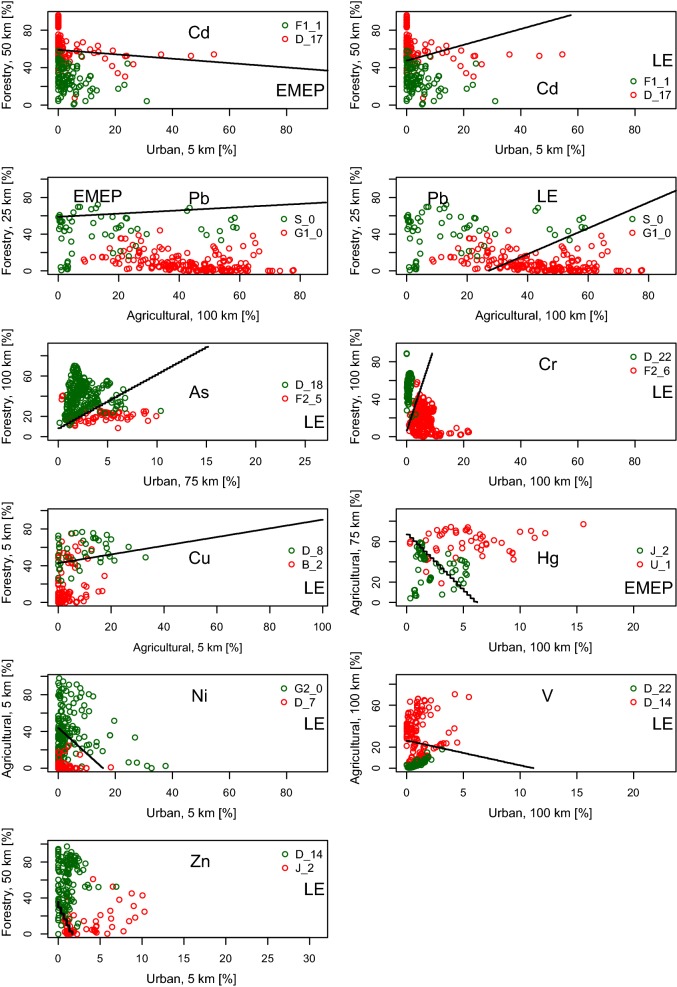
Discriminant functions of LDA models for As, Cd, Cr, Cu, Hg, Ni, Pb, V, and Zn. Discriminant functions (black lines) with relevant densities of land use around the sampling sites as predictors for separating between two ELCE revealing extremely high (= green) and low (= red) correlations selected as examples; atmospheric deposition was modelled by LE (2009–2011) or EMEP (2008–2010)

The frequency of the predictors used as discriminants in the 11 LDA models ranges between 1 and 3, which means that none of the factors in particular stands out (Fig. 4). Moreover, the relevance of the predictors for separating sampling sites contributing to high or low correlations is element specific. When taking areal percentage of urban and agricultural land use as indicators for potential influences of areal and point emission sources, small radiuses around the sampling sites (5 km) are obviously more relevant for Cd, Cu, Ni, and Zn than that for the other elements examined. Vice versa, areal percentage of urban and agricultural land use within large radiuses (75–100 km) is more relevant for As, Cr, Hg, Pb, and V.

LDA models with the highest quality corresponding to error rates ≤ 30% were found for Cr and V followed by As, Cu, Hg, and Pb (only LE) with error rates ≤ 40% (Table 3), i.e. in 7 out of 11 cases < 40% of the sampling sites has been incorrectly classified according to their surrounding land use. Although all predictors were not normally distributed, which is a fundamental assumption for LDA, error rates of the logistic regression models (LR) using the same predictors as the LDA models were very similar.

**Table 3:** Error rates of LDA and LR models specified for nine heavy metals and two chemical transport models

	EMEP			LOTOS-EUROS							
	Cd	Hg	Pb	As	Cd	Cr	Cu	Ni	Pb	V	Zn
LDA (%)	41	*34*	42	*36*	44	*29*	*39*	43	*34*	*26*	41
LR (%)	41	*33*	42	*36*	44	*30*	*39*	43	*34*	*26*	41

Error rates of best models (< 40%) are in italic print; atmospheric deposition was modelled by LE (2009–2011) or EMEP (2008–2010)

From Tables 2 and 3, it is obvious that LDA models are appropriate, particularly in case of elements showing low correlations between atmospheric deposition and concentrations in moss (Cr, Cu, Hg, V). For Cd and Pb with strong correlations, density of land use around the sampling sites seems to be less relevant. This is also confirmed by the statistical indicators for the significance of the predictors given from LR modelling: Density of urban land use (5 km) for Cd (EMEP, LE) and agricultural land use (100 km) as a predictor for Pb (EMEP) was both non-significant, which may also explain the high error rates of 41–44%.

Figure 4 shows the discriminant lines obtained from LDA. The 11 scatter plots exemplify the separation between sampling sites contributing to high and low correlation. Since the whole set of ELCE would lead into non-readable graphs, ELCE units with maximum and minimum correlation coefficients have been selected as examples. Error rates of 26–44% (Table 3) are reflected in discriminant lines not really separating green and red points. Resulting from this, LDA models for As, Cr, Cu, Hg, Pb, and V prove to be the most appropriate. The location of point clusters in case of Ni and Zn appears to be implausible, because low densities of urban and agricultural land use around the sampling sites should result in a higher and not in a lower correlation (Ni), and discriminant lines between forestry and urban land use should in principal reveal a positive slope (Zn), as can be seen for As, Cr, Cu, and Pb. This may also explain the high error rates of 41–43% in case of Ni and Zn.

Table 4 shows which LDA models are feasible for predicting correlation levels depending on land use around the sampling sites: The subset of A sites selected from the whole dataset by LDA (row 2) reveals increased correlation coefficients in case of Hg, As, Cr, Cu, and V compared to the European dataset, despite a smaller sampling size. Regarding the subsample of sites within ELCE regions showing above-average correlations (rows 4–6), sampling sites classified to B (row 6) reveal remarkably lower correlations for Cd, Pb, and Cr, and sites classified to A (row 5) remarkably higher correlations for Cr compared to the whole partial sample (row 4). The same effect of increasing (row 8) and decreasing correlations (row 9) is visible also for the sampling sites within ELCE B regions (rows 7–9) with respect to the LDA modelling results for Hg, As, Cr, Cu, Ni, Pb, and V.

**Table 4:** Correlations between heavy metal concentrations in moss and atmospheric deposition specified for ELCE categories and sampling site categories

Row ID	ELCE category	Site category	EMEP			LOTOS-EUROS							
			Cd	Hg	Pb	As	Cd	Cr	Cu	Ni	Pb	V	Zn
1	All	All	0.65**	0.14**	0.70**	0.30**	0.65**	0.03*	0.50**	0.09**	0.64**	0.19**	0.17**
2	All	A	0.66**	0.24**	0.70**	0.42**	0.62**	0.44**	0.62**	0.15**	0.67**	0.28**	0.17**
3	All	B	0.41**	− 0.05**	0.46**	0.09**	0.60**	− 0.29**	0.40**	− 0.20**	0.44**	*0.01*	− *0.02*
4	A	All	0.75**	0.22**	0.72**	0.45**	0.72**	0.22**	0.50**	0.16**	0.67**	0.21**	0.22**
5	A	A	0.77**	0.22**	0.74**	0.44**	0.72**	0.49**	0.52**	0.19**	0.66**	0.21**	0.22**
6	A	B	0.26**	0.17	0.28	0.35**	0.45**	− 0.15**	0.51**	− 0.08**	0.60**	0.14**	0.06
7	B	All	0.50*	0.06	0.67**	0.07**	0.58**	− 0.26**	0.45**	− 0.04**	0.44**	0.13**	0.10**
8	B	A	0.47**	0.27**	0.62**	0.32**	0.48**	0.15**	0.65**	0.09**	0.54**	0.33**	0.10**
9	B	B	0.54**	− 0.18**	0.55**	− 0.10**	0.67**	− 0.36**	0.27**	− 0.34**	0.27**	− 0.02	− 0.11

ELCE category: All = ELCE regions as a whole; A = ELCE regions showing correlations above the element-specific class boundary between high and low correlation levels given in Table 2; B = ELCE regions showing correlations below the element-specific class boundary between high and low correlation levels given in Table 2; site category: All = Moss sampling sites as a whole; A = moss sampling sites classified by LDA model as A; B = moss sampling sites classified by LDA model as B; correlation coefficients according to Spearman; significant correlations are in italic print (*p < 0.05, **p < 0.01); atmospheric deposition was modelled by LE (2009–2011) or EMEP (2008–2010)

Based on the study results described above, LDA models for Pb (LE), As, Cr, Cu, Hg, and V were selected as the most valid models further pursued for predictive mapping of correlations in “Predictive mapping of correlations” section.

### Predictive mapping of correlations

**Fig. 5:**
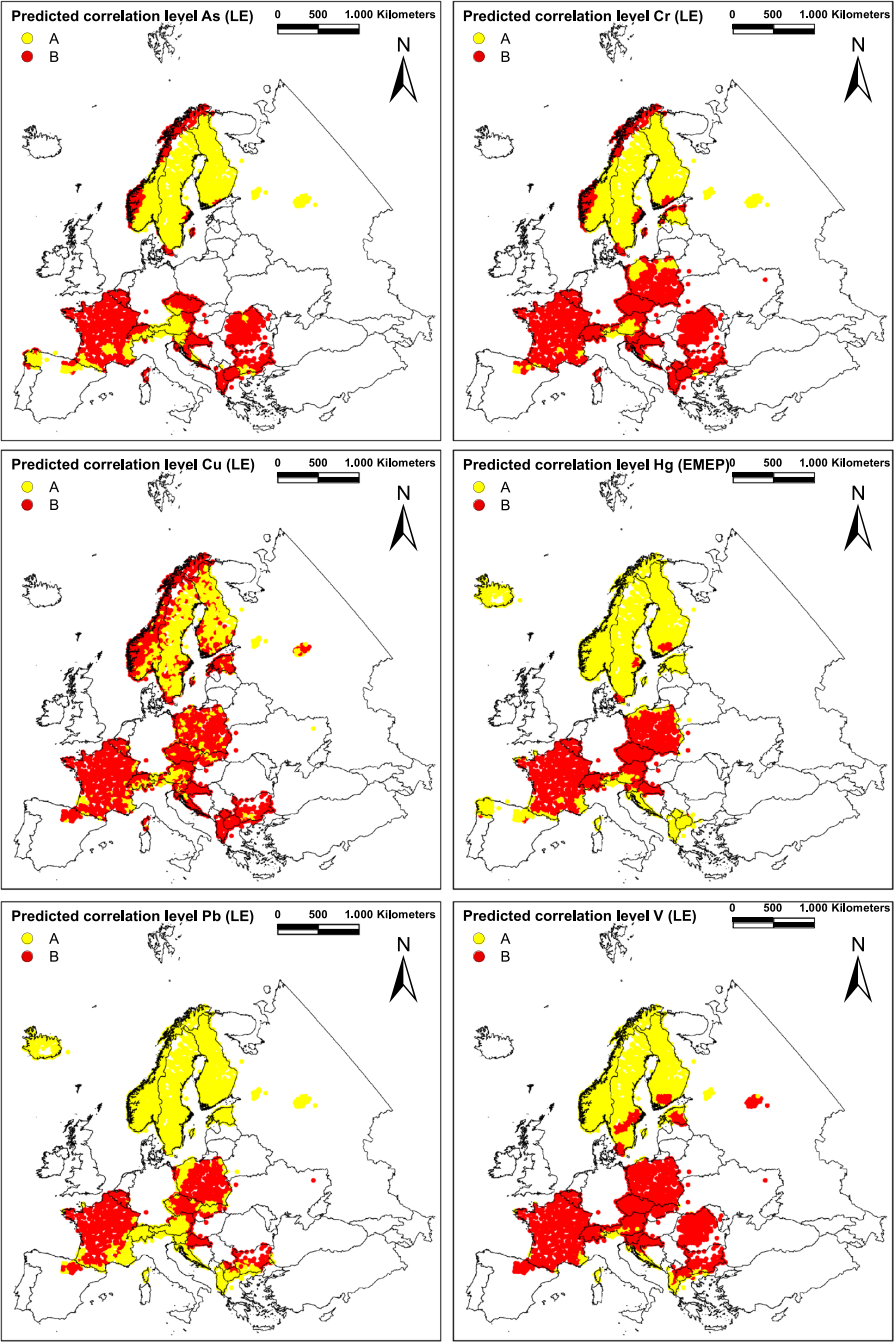
Predicted correlation patterns for As, Cr, Cu, Hg, Pb, and V (moss; EMEP/LE) at site level as classified according to their surrounding land use with above element-specific average (= A) or below-average (= B) correlations (cf. Table 2)

The application of LDA models for As, Cr, Cu, Hg, Pb (LE), and V on the whole European dataset with information on density of land use around the sampling sites led to typical correlation patterns as depicted in Fig. 5. While northern Europe and the Alps are predominately characterized by correlations above the average, the other regions frequently reveal low correlation levels. Further correlation patterns for the remaining element are presented in Additional file 1: Figure S2.

**Fig. 6:**
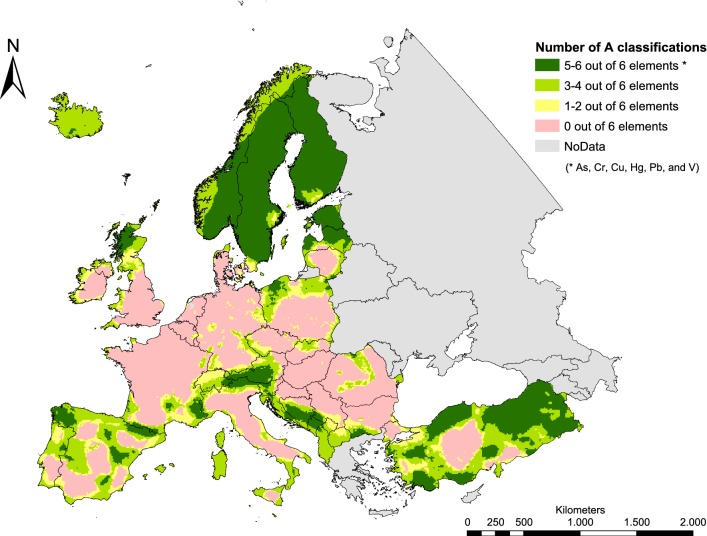
Number of A classifications as modelled by LDA for As, Cr, Cu, Hg, Pb, and V

An aggregated view on LDA predictions for As, Cr, Cu, Hg, Pb, and V feasible within countries supplying sufficient information on LDA predictors with blanket coverage derived from CLC 2006 is shown in Fig. 6.

Areas with 5–6 out of 6 LDA models revealing potential high correlations are located in Fennoscandia, Scotland, Austria and parts of the Baltics, South-eastern Europe, Turkey, France, and Spain (= 20% of the area covered with spatial information on land use density around 10 km × 10 km grids). Information about the elements being relevant at specific locations is given in Fig. 5. Countries comprising regions with consistently low potential correlations (0 out of 6 classifications to A) can be in particular found in western, Central and South-eastern Europe (= 38% of the area being investigated). The remaining area (42%) is characterized by locations where correlation levels for 1–4 out of 6 elements were classified to A.

## Discussion

### Correlation analysis

For all investigated HM (As, Cd, Cr, Cu, Hg, Ni, Pb, V, and Zn), the correlations between concentrations in moss and the modelled total atmospheric deposition (EMEP, LOTOS-EUROS) were land class specific and element specific. Significant positive correlations were found for about 30% (As), 78–81% (Cd), 15% (Cr), 63% (Cu), 44% (Hg), 15% (Ni), 78–89% (Pb), 19% (V), and 44% (Zn) of 27 ELCE units being represented by moss sample sizes *n* > 10. This is in line with similar findings for Cd, Hg and Pb based on data from 2005 [9, 10] and was supplemented for further seven elements. This study confirms that mosses are good biomonitors for atmospheric deposition of Cd and Pb [38] and to some extent also for Cu, but this study suggests that mosses are less good as biomonitors for other HM like Cr, Hg, Ni, V, and Zn. The amount of ELCE-specific correlation coefficients is closely connected to uncertainties in deposition modelling contributing to variation and potential influencing factors in the use of mosses as monitors of atmospheric deposition [38, 39]. Particular mention is to be made of different emission data used for EMEP and LE [40] probably explaining the differences in the correlations (Table 2) and, respectively, the direction and/or intensity of the discriminant lines (Cd and Pb in Fig. 4). Besides this, modelled data are grid-specific (LE: 25 km × 25 km; EMEP: 50 km × 50 km) and moss data are site specific, meaning that we are always working at spatial limits of extrapolation in the moss monitoring network. Density of land use around the sampling sites should not only considered as a relevant factor for element concentrations in moss [12, 13], but even more as an indicator for influencing factors such as areal and point emission sources that are very evident in some Balkan countries effected mainly from windblowing dust and mineral particles [41–43]. Further important factors than those considered in the modelling of background atmospheric deposition influencing the amount of correlation are particularly mineral particles, mainly windblown dust from local soil (As, Cd, Cr, Cu, Hg, Ni, V) and root uptake in higher plants from soil and transfer to mosses by leaching from dead or living plant material [44]. Low correlations with As implies that As metabolism could be more involved than expected [45]. Also precipitation pH may play a role because it affects the solubility of heavy metals and hence their uptake by mosses which should be taken into account in further investigations. Due to the existing uncertainties in the deposition modelling, future studies should also include comparisons between heavy metal concentrations in moss tissue and atmospheric deposition measured by technical samplers, e.g. within the EMEP framework [22]. Also, other potential pollutants, such as nitrogen or persistent organic pollutants (POP), should be included. First pilot studies on POP exist, for example, in Germany [5].

### Statistical modelling

The use of LDA enables a separation of moss sampling sites regarding their contribution to the correlation level in dependency on urban, agricultural and forestry land use around the sampling location. LDA and LR help explaining spatial patterns of ELCE-specific correlation levels by identifying most relevant land uses and radiuses around the sampling sites. A very valuable indicator for this was that moss sampling sites classified by LDA as A within ELCE B units defined by below element-specific average correlations show stronger correlations than the whole set of sampling sites within ELCE B units (Hg, As, Cr, Cu, Pb, V). Vice versa, category B sites from ELCE A units reveal lower correlations than the whole subsample from ELCE A units (As, Cd, Pb, Cr, Ni). This means that the ELCE-specific correlation levels are more strongly dependent on land use within the specified radii around the sampling sites and less on the spatial assignment to specific ELCE units, which per se have high or low correlations. For example: Each random subsample within regions showing low correlations between Hg concentrations in moss and respective atmospheric deposition is expected to reveal low correlations. If LDA selects a subsample indicating high correlations within regions showing low correlations, the importance of the predictors for Hg can be graded as high. Best LDA models with error rates < 40% were found for As, Cr, Cu, Hg, Pb, and V. Similar error rates were found by means of LR, confirming the validity of LDA models, which are more graphic and less difficult to interpret. This supports our approach to use LDA in conjunction with a confirmation by LR, which is in any case recommended for multivariate statistics, especially since predictors are not normally distributed. Likewise, relevant factors for predicting potential correlation levels are element specific. While density of forestry land use might characterize a seclusion of sampling locations, urban and agricultural land use around the sampling sites might indicate pronounced influences of local emission sources. For Cd, Cu, and Ni, small radiuses are relevant. For Cd and Cu, the relevance of agricultural land use within a small range of 0–5 km could be caused by fertilizers (Cd) and copper-containing pesticides [46]. For Cd and Ni, the relevance of urban land use within a radius of 5 km might be connected to local industrial emission sources. By contrast, large radiuses of 75–100 km are most relevant for As, Hg, Cr and V, which predominantly show the weakest statistical relations to modelled atmospheric deposition. At least for Hg, this could be a result of long residence times of Hg components in the atmosphere and correlated long-range transport [47, 48]. Regarding As and Cr, high density of forestry land use within a 100 km radius, which might indicate a low influence of local emission sources, seems to be a crucial factor. Regarding As, Hg, Cr and V, this study indicates that the radius for examining the influence of different spatial land use density around the sampling sites could be even more enlarged (e.g. 150, 200 or 250 km) for a probably better consideration of the long-range transboundary air pollution.

### Predictive mapping

LDA modelling in combination with LR is an eligible method for predicting and mapping spatial patterns of correlations in dependency of the influence of the environmental factors. Based on error rates and spatial patterns analysis, the explanatory power of 6 out of 11 LDA models with error rates < 40% (As, Cr, Cu, Hg, Pb, and V) was found to be sufficient. However, error rates of 26–39% imply strong influences of other factors, i.e. the predictions are connected with high uncertainties and should be regarded as indications only.

## Conclusions

The predictive mapping provides spatial indication on the presence of other factors for element concentration in moss than those considered in the modelling of background atmospheric deposition, e.g. local emission sources or mineral windblown dust from soil [44]. The LDA discriminants could be, at least on the large scale, used as additional criteria for planning moss survey networks beyond the recommendations of the Moss Manual such as minimum distances from roads or houses [19]. Besides, the classification and selection of sampling sites can serve as a preliminary stage for detailed site-specific investigations of possible local factors. To this end, binary classification (A, B) of the LDA models should be refined, in order to increase the spatial differentiation in particular for countries revealing low predicted correlation levels (e.g. Belgium, northern France). The predicted correlation level could then be integrated into more comprehensive sets of criteria for planning (reductions of existing) moss survey networks [49]. Predictive maps could also be useful for initial estimations of the correlation level in regions beyond the EMS network having sufficient information on land use with blanket coverage (e.g. Estonia, Kosovo, parts of Spain or Turkey). The underlying assumption is that land use around potential sampling sites in countries that did not submit data for 2010 EMS affects the correlation on the same order of magnitude as in countries that participate in EMS. For decision support, it is recommended to aggregate models results to reduce complexity. LDA models and predictive mapping should be validated with further independent data from the EMS 1990, 1995, 2000, 2005, and 2015.

## Additional file

**Additional file 1: Figure S1.** Map of Ecological Land Classes of Europe [15]. **Table S1.** Legend of the map on Ecological Land Classes of Europe [15]. **Table S2.** Description of Chemical Transport Models (CTM) used as data source. **Figure S2.** Predicted correlation patterns for Cd, Ni, Pb, and Zn (moss; EMEP/LE) at site level as classified according to their surrounding land use with above element-specific average (= A) or below e.-s. average (= B) correlations.

## Abbreviations

As: arsenic
B_1: western and northern Scandinavia, Northwest Russia
B_2: the Alps, Iceland, Northwest Russia
C_0: the Alps, Iceland, western and northern Scandinavia, Kola Peninsula, Northwest Russia, Caucasus
Cd: cadmium
CLRTAP: convention on long-range transboundary air pollution
Cr: chromium
CTM: chemical transport model
Cu: copper
D_10: Russia
D_13: the Alps, dispersed small areas in eastern and Southeast Europe
D_14: Baltic states, Belarus, western Russia
D_16: Northeast European hygrophilous spruce forests with dwarf scrubs, sedges and mosses
D_17: Scandinavia, western Russia
D_18: southern Scandinavia, northern Baltic states
D_19: southern/central Finland, Norway
D_21: Northwest Russia
D_22: Sweden, Northwest Russia
D_7: Scandinavia, Northwest Russia
D_8: Kola Peninsula, Northwest Russia
ELCE: ecological land classes of Europe
EMEP: European monitoring and evaluation programme
EMS: European Moss Survey
F1_1: Poland, Northwest Ukraine
F1_2: Ireland, Great Britain, western and central Europe
F2_5: southern Baltic states, eastern Poland, western and Southwest Ukraine
F2_6: central Europe, eastern and Southeast Europe
F3_1: Germany, Northwest Poland, Czech Republic, northern Austria, Slovenia, the Balkans
F3_2: western Europe (including northern Spain, France, Benelux countries, western Germany), Denmark
F4_1: Southeast Great Britain, Southeast Denmark, Northeast Germany, Northwest Poland
F4_2: western/central and southern Europe (including southern Great Britain, eastern France, southern Belgium, Luxembourg, the Alps, Italy), eastern and Southeast Europe (including the Carpathian Mountains, the Balkans)
G1_0: Italy, Southeast Europe
G2_0: Iberian Peninsula, southern and Southeast Europe
Hg: Mercury
HM: heavy metals
ICP: international cooperative programme
J_2: Iberian Peninsula, coastal areas by the Mediterranean Sea
L_2: eastern Europe (Hungary, Romania, Moldova, Ukraine, Russia)
LDA: linear discriminant analysis
LE: LOTOS-EUROS
LR: logistic Regression
M_5: eastern Ukraine, Southwest Russia, Caukasus
M_6: eastern Romania, southern Ukraine
MSC-E: meteorological synthesizing centres MSC-East (Moscow)
N: nitrogen
Ni: nickel
Pb: lead
POP: persistent organic pollutants
*r*_s_: correlation coefficient (Spearman)
S_0: northern parts of Europe (including parts of Iceland, Ireland, Great Britain, Scandinavia, Northwest Russia, the Blatic states and Belarus)
U_1: dispersed small areas within a stripe reaching from Ireland via central Europe and the Byelorussian-Ukrainian borderline to Russia
U_2: dispersed small areas in southern Europe reaching from the Iberian Peninsula via Southeast Europe including, e.g. the Balkans, the Carpathians, Greece and northern Turkey to Southwest Russia
UN-ECE: United Nations Economic Commission for Europe
V: vanadium
Zn: zinc
