# Supplementary material for: Modelling spatial patterns of correlations between concentrations of heavy metals in mosses and atmospheric deposition in 2010 across Europe
Source: Environ Sci Eur. 2018 Dec 21;30(1):53. doi: 10.1186/s12302-018-0183-8 (PMC6302881; doi:10.1186/s12302-018-0183-8)

Additional file 1:

Figure S1. Map of Ecological Land Classes of Europe (Hornsmann et al. 2008) (Legend: **Table S1**)

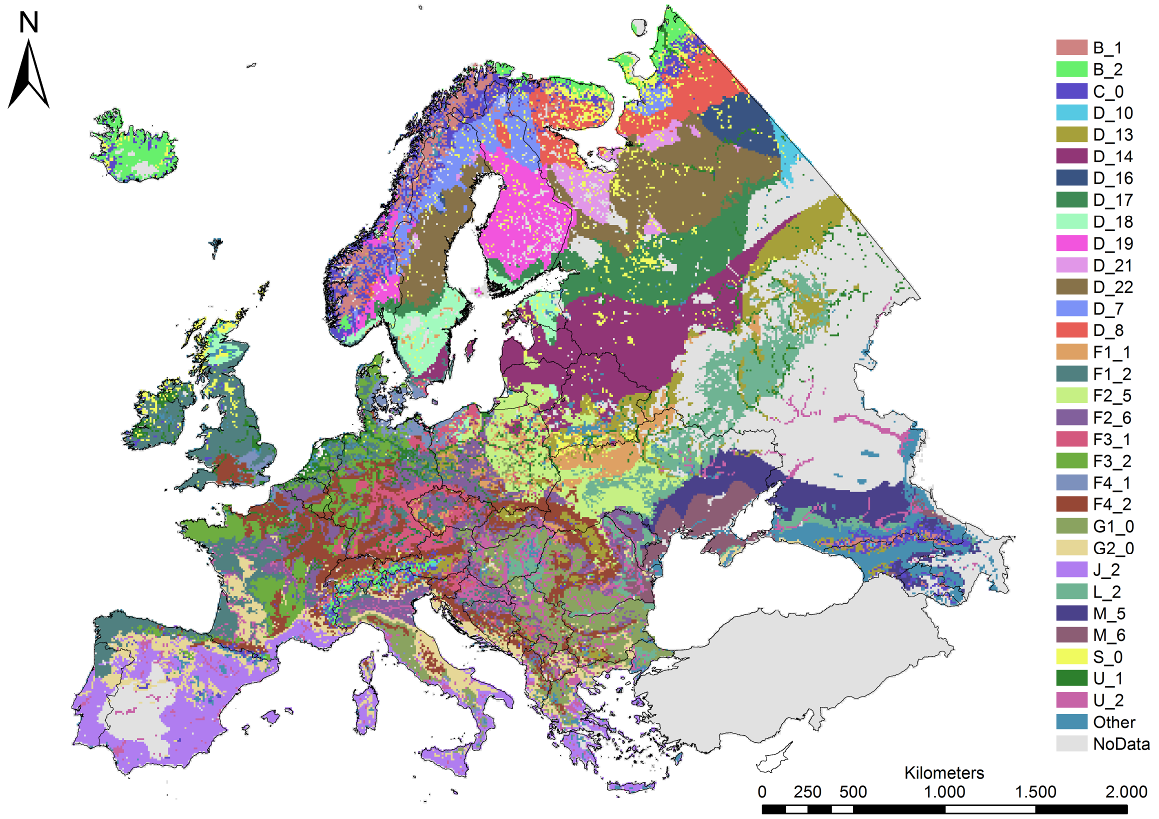

Table S1. Legend of the map on Ecological Land Classes of Europe (Hornsmann et al. 2008)

ELCE	Main distribution	Elev. [m.a.s.l].	Prec. [mm/month]	Area [km²]
**B_1**	Western and northern Scandinavia, northwest Russia	918	71	178800
**B_2**	The Alps, Iceland, northwest Russia	577	74	311400
**C_0**	The Alps, Iceland, western and northern Scandinavia, Kola Peninsula, northwest Russia, Caucasus	846	79	321800
**D_7**	Scandinavia, northwest Russia	341	46	185700
**D_8**	Kola Peninsula, northwest Russia	209	48	287800
**D_10**	Russia	481	62	96700
**D_13**	The Alps, dispersed small areas in eastern and southeast Europe	415	57	377500
**D_14**	Baltic States, Belarus, western Russia	146	52	537900
**D_16**	Northeast European hygrophilous spruce forests with dwarf scrubs, sedges and mosses	166	52	89800
**D_17**	Scandinavia, western Russia	166	50	336000
**D_18**	Southern Scandinavia, northern Baltic States	132	54	150500
**D_19**	Southern/central Finland, Norway	128	64	227100
**D_21**	Northwest Russia	183	54	92800
**D_22**	Sweden, northwest Russia	120	47	534000
**F1_1**	Poland, northwest Ukraine	185	52	162000
**F1_2**	Ireland, Great Britain, western and central Europe	190	80	431000
**F2_5**	Southern Baltic States, eastern Poland, western and southwest Ukraine	192	51	231400
**F2_6**	Central Europe, easterm and southeast Europe	247	60	345200
**F3_1**	Germany, northwest Poland, Czech Republic, northern Austria, Slovenia, the Balkans	466	67	154800
**F3_2**	Western Europe (including northern Spain, France, Benelux countries, western Germany), Denmark	302	69	225100
**F4_1**	Southeast Great Britain, southeast Denmark, northeast Germany, northwest Poland	135	53	79900
**F4_2**	Western/central and southern Europe (including southern Great Britain, eastern France, southern Belgium, Luxembourg, the Alps, Italy), eastern and southeast Europe (including the Carpathian Mountains, the Balkans)	589	73	483300
**G1_0**	Italy, southeast Europe	354	57	303000
**G2_0**	Iberian Peninsula, southern and southeast Europe	572	69	296200
**J_2**	Iberian Peninsula, coastal areas by the Mediterranean Sea	457	53	438200
**L_2**	Eastern Europe (Hungary, Romania, Moldova, Ukraine, Russia)	186	47	352500
**M_5**	Eastern Ukraine, Southwest Russia, Caukasus	292	44	233800
**M_6**	Eastern Romania, southern Ukraine	110	39	131100
**S_0**	Northern parts of Europe (including parts of Iceland, Ireland, Great Britain, Scandinavia, northwest Russia, the Blatic states and Belarus)	162	60	271600
**U_1**	Dispersed small areas within a stripe reaching form Ireland via central Europe and the Byelorussian-Ukrainian borderline to Russia	111	56	199300
**U_2**	Dispersed small areas in southern Europe reaching form the Iberian Peninsula via southeast Europe including e.g. the Balkans, the Carpathians, Greece and northern Turkey to southwest Russia	174	53	239900
**Others**	Southwest Russia, Georgia, Azerbaijan, Armenia and further small areas all across Europe	585	65	347800

ELCE = 31 Ecological land classes and other ELCE which were summarized to one class (“Others”);

Table S2. Description of Chemical Transport Models (CTM) used as data source

Data source	Description
EMEP MSCE-HM model	*Regional heavy metal transport model (MSCE-HM)*<br>Developer: Meteorological Synthesizing Centre - East (MSC-E)<br>Model type: Eulerian 3D grid model<br>Input: Atmospheric emissions, meteorological information, geophysical data (land cover data, leaf area index, oceanological input data)<br>Output: Heavy metals (Cd, Pb, Hg)<br>Domain: 35° West to 60° East and from the North Pole to about 20° North<br>Spatial resolution: 50 x 50 km (Europe)<br>References: Travnikov O., Ilyin I. (2005) Regional Model MSCE-HM of Heavy Metal Transboundary Air Pollution in Europe. EMEP/MSC-E Technical Report 6/2005, p.59.<br>Tørseth K, Aas W, Breivik K, Fjæraa M, Fiebig M, Hjellbrekke, AG, Lund Myhre C, Solberg S, Yttri KE (2012) Introduction to the European Monitoring and Evaluation Programme (EMEP) and observed atmospheric composition change during 1972-2009. Atmos Chem Phys 12:5447-5481
LOTOS-EUROS (*LE*)	*LOng Term Ozone Simulation - EURopean Operational Smog model*<br>Developer: TNO Institute of Environmental Sciences, Utrecht, Netherlands<br>Model type: Eulerian 3D grid model<br>Input: Anthropogenic emissions, 3-hourly meteorological data, topography (tree species coverage, land cover data)<br>Output: Oxidants (O_3, VOCs, NO_x, HNO_3 etc.); Secondary Inorganic Aerosol (SO_4, NO_3, NH_4); Secondary Organic Aerosols (SOA) from terpenes; Primary aerosol ($PM_{2.5}$; PM_{10}, Black Carbon, sea salt); Heavy metals (Cd, Pb and other non-volatiles metals); Persistant Organic Pollutants (POPs)<br>Domain: 35° and 70° North and 10° West and 60° East<br>Spatial resolution: 25 x 25 km (Europe); 7 x 7 km (national level)<br>References: Schaap, M., Roemer, M., Sauter, F., Boersen, G., Timmermans, R., Builjes, P.J.H., Vermeulen, A.T. (2005) LOTOS-EUROS: Documentation, TNO report B&O-A R 2005/297.<br>Schaap, M., Timmermans, R., Roemer, M., Boersen, G., Builjes, P.J.H. (2008) The LOTOS–EUROS model: description, validation and latest developments. Int. J. Environment and Pollution, Vol. 32, No. 2:270-290.European Topic Centre on Air and Climate Change (ETC/AAC) (2010) Long description of model 'LOTOS-EUROS. http://pandora. meng.auth.gr/mds/showlong.php?id=57 (23.11.2015).

Figure S2. Predicted correlation patterns for Cd, Ni, Pb, and Zn (moss; EMEP / LE) at site level as classified according to their surrounding land use with above element-specific average (= A) or below e.-s. average (= B) correlations.

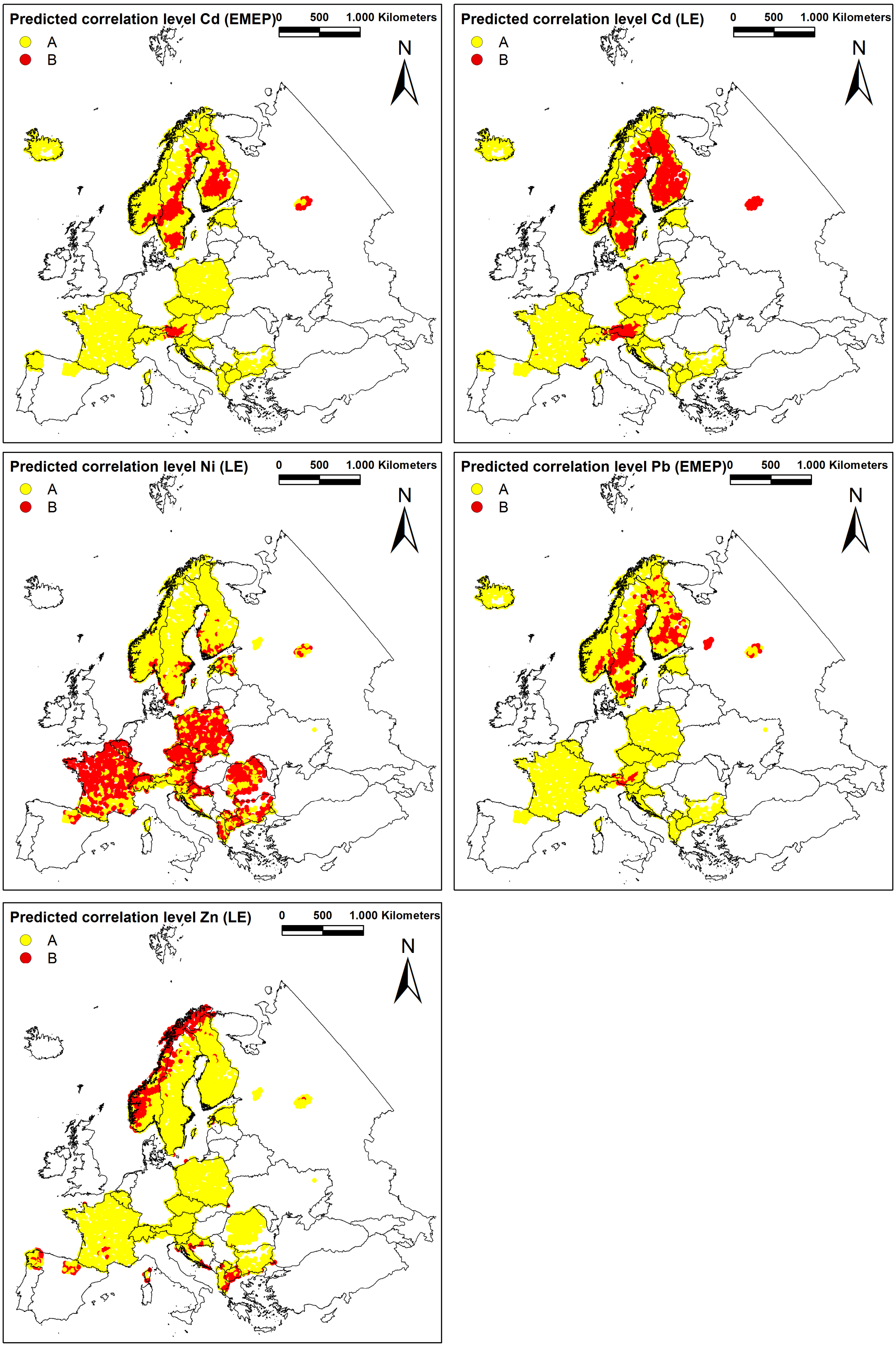

Supplement: Supplementary file 1 — Additional file 1: Figure S1. Map of Ecological Land Classes of Europe [15]. Table S1. Legend of the map on Ecological Land Classes of Europe [15]. Table S2. Description of Chemical Transport Models (CTM) used as data source. Figure S2. Predicted correlation patterns for Cd, Ni, Pb, and Zn (moss; EMEP/LE) at site level as classified according to their surrounding land use with above element-specific average (= A) or below e.-s. average (= B) correlations. [file 12302_2018_183_MOESM1_ESM.pdf]
